# Targeting Hsp20 Using the Novel Small Non-coding RNA *DnrH* Regulates Heat Tolerance in *Deinococcus radiodurans*

**DOI:** 10.3389/fmicb.2019.02354

**Published:** 2019-10-11

**Authors:** Dong Xue, Yun Chen, Jiang Li, Jiahui Han, Yingying Liu, Shijie Jiang, Zhengfu Zhou, Wei Zhang, Ming Chen, Min Lin, Marc Ongena, Jin Wang

**Affiliations:** ^1^Biotechnology Research Institute, Chinese Academy of Agricultural Sciences, Beijing, China; ^2^Microbial Processes and Interactions, TERRA Teaching and Research Centre, Gembloux Agro-Bio Tech, University of Liège, Gembloux, Belgium; ^3^Department of Plant Science, School of Agriculture and Biology, Shanghai Jiao Tong University, Shanghai, China; ^4^College of Life Science and Engineering, Southwest University of Science and Technology, Mianyang, China

**Keywords:** small non-coding RNA, *Deinococcus radiodurans*, heat stress, *DnrH*, *Hsp20* mRNA

## Abstract

Small non-coding RNAs (ncRNAs) are a class of regulatory molecules, which remain understudied in bacteria. In the extremophilic bacterium *Deinococcus radiodurans*, although hundreds of ncRNAs have been identified, few have been characterized in detail. In this study, we report the identification and characterization of a novel heat-inducible ncRNA named *DnrH*. Heat tolerance analysis showed that deleting *DnrH* significantly inhibited viability in response to high temperature conditions. Comparative phenotypic and qRT-PCR analyses of a *DnrH* mutant (Δ*DnrH*) and wild-type (WT) *D. radiodurans* suggested that *DnrH* is potentially involved in regulating the expression of the heat shock-related gene *Hsp20*. Microscale thermophoresis and genetic complementation showed that a 28-nucleotide (nt) sequence in the stem-loop structure of *DnrH* (143–170 nt) pairs with its counterpart in the coding region of *Hsp20* mRNA (91–117 nt) via a 22 nt region. *In vivo*, mutation of the 22-nt region in the *D. radiodurans* genome led to a reduction in heat tolerance similar to that observed in the *DnrH*-mutant. Our results show that *DnrH* positively influences heat tolerance by increasing the transcription of *Hsp20* mRNA, demonstrating, for the first time, a ncRNA that directly controls the expression of a heat stress-resistance gene. This work provides new insight into the heat stress response mechanism of *D. radiodurans* as well as other extremophiles that express similar Hsp20 proteins.

## Introduction

Small non-coding RNAs (ncRNAs) play critical roles in gene expression at the posttranscriptional level and are recognized as key transcriptional regulators in bacteria (Liu and Camilli, 2010; Arnvig et al., 2011; Acebo et al., 2012). Generally, ncRNAs remain untranslated with typical lengths ranging from approximately 50 to 500 nucleotides (nt) (Wassarman, 2002). Over the past decades, many ncRNAs have been identified both in gram-positive and gram-negative bacteria (Wassarman et al., 1999; Del Val et al., 2007; Mandin et al., 2007; Landt et al., 2008; Arnvig et al., 2011; Acebo et al., 2012) and these have been divided into four groups as follows: *cis*-encoded, *trans*-encoded, protein-binding, and CRISPR ncRNAs. *Trans*-encoded ncRNAs are the best characterized and most extensively studied (Wagner and Romby, 2015) and exert their regulatory functions through imperfect base-pairing to modulate target mRNA stability and/or translation (Richards and Vanderpool, 2011; Storz et al., 2011). Nonetheless, a wide range of physiological functions may be regulated by ncRNAs, many of which are related to environmental changes, such as iron limitation, acidity, osmotic shock, envelops stress, temperature, and nutrient stress (Lybecker and Samuels, 2007; Jin et al., 2009; Patel et al., 2009; Boysen et al., 2010; Fantappiè et al., 2011; Fröhlich et al., 2012; Barreto et al., 2016).

*Deinococcus radiodurans* is a model species best known for its extraordinary resistance to diverse environmental stress factors, such as ionizing radiation, ultraviolet irradiation, desiccation, oxidation, and temperature (Blasius et al., 2008; Wang et al., 2008; Daly, 2009; Patel et al., 2009; Bauermeister et al., 2011; Timmins and Moe, 2016). Because of its genetic operability and extreme resistance, *D. radiodurans* thus represents a leading model to study various extreme stress environments. Temperature fluxes have been reported to be a vital factor for bacterial life, affecting metabolism, and protein unfolding and aggregation in microbes and leading to a higher risk of cell death (Lesley et al., 2002; Ventura et al., 2006). Studying the regulatory mechanisms involved in temperature stress responses of *D. radiodurans* may provide new insights into the stress response of other unrelated species. Multiple genes associated with heat tolerance have been identified in *D. radiodurans* and the first study of heat stress in *D. radiodurans* was published in 1988. Results from this study suggested that proteins synthesized *de novo* during incubation at different temperature intervals (exposure to 52°C for 30 min, with immediate subsequent transfer to 30°C or 42°C for various intervals) are involved either in the thermotolerance phenomenon itself or the recovery from injury (Harada and Oda, 1988). In the year following the aforementioned study, various heat-inducible proteins were discovered (generally, by proteomics analyses), including Hsp20, GroEL, DnaK, SodA, Csp, and Protease I (Airo et al., 2004; Schmid et al., 2005c). Sig1 was determined to be essential for induction of the heat shock proteins groESL and dnaKJ (Schmid and Lidstrom, 2002; Schmid et al., 2005a), whereas *hspR* binds HAIR sites in close proximity to promoter regions, thereby directly inhibiting the expression of regulated genes encoding chaperone proteins and proteases (Schmid et al., 2005b). Recently, an analysis of two small heat shock proteins (sHSPs; IbpA, and IbpB) showed that in *D. radiodurans*, these proteins are very different in term of their quaternary structures and chaperone properties and were considered to represent a second type of bacterial two-component sHsp systems (Bepperling et al., 2012). More recently, DdrI (encoded by DR_0997) was reported enhance heat tolerance in *D. radiodurans* (Meyer et al., 2018).

Using a genome-wide RNA sequencing approach, Tsai et al. (2015) identified 41 ncRNA candidates; however, functional characterizations of these molecules are still lacking. Considering that ncRNAs often act as regulators in response to various stresses, it is plausible that they could play important roles in the extremophilic properties of *D. radiodurans*. In this study, we describe the function and targets of a novel ncRNA that is potentially involved in heat tolerance in *D. radiodurans*. This ncRNA was designated *DnrH* (*D. radiodurans* ncRNA response to heat stress) and its expression was found to be upregulated during heat stress. Further characterization and target identification showed that *DnrH* functions by directly binding *Hsp20* mRNA, which provides evidence of a novel posttranscriptional regulatory mechanism underlying the heat stress response of *D. radiodurans*.

## Materials and Methods

### Bacterial Strains, Plasmids, Primers, and Culture Conditions

All strains and plasmids used in this study are described in Table 1. *D. radiodurans* was obtained from China General Microbiological Culture Collection Center (CGMCC 1.633, Beijing, China). *D. radiodurans* and derivatives were routinely cultured in TGY broth (1% tryptone, 0.5% yeast extract, and 0.1% glucose) or on TGY plates supplemented with agar (1.5%) at 30°C. *Escherichia coli* strains were grown in Luria-Bertani (LB) broth or on LB plates supplemented with agar (1.5%) at 37°C. When required, ampicillin, kanamycin, spectinomycin, and chloromycetin were added to final concentrations of 50, 20, 340, and 3.4 μg/mL, respectively.

**TABLE 1:** Strains and plasmids used in this study.

**Strain/plasmid**	**Relevant characteristics**	**Source**
***Deinococcus radiodurans***	WT	Lab stock
Δ*DnrH*	*D. radiodurans* DnrH-deletion mutation, kanamycin	This study
*Com-DnrH*	*D. radiodurans* DnrH-deletion mutation containing the complementation plasmid pRADZ3-DnrH, kanamycin, and chloromycetin	This study
Δ*Hsp20*	*D. radiodurans* Hsp20-deletion mutation, spectinomycin	This study
*cHsp20*	*D. radiodurans* containing the complementation plasmid pRADZ3-Hsp20, spectinomycin and chloromycetin	This study
*cDnrH-mut*	*D. radiodurans* DnrH-deletion mutation containing the complementation plasmid pRADZ3-DnrH-mut, kanamycin and chloromycetin	This study
***Escherichia coil***		
*Trans 109*	Host for plasmid sub-cloning	TransGen
*Trans 109 Z3*	As trans109 with pRADZ3	This study
*Trans 109 Z3-DnrH*	As trans109 with Z3-DnrH	This study
*Trans 109 Z3-Hsp20*	As trans109 with Z3-Hsp20	This study
*Trans 109 Z3-DnrH-mut*	As trans109 with Z3-DnrH-mut	This study
**Plasmid**		
*pRADZ3*	Shuttle plasmid between *E. coli* and *D. radiodurans*, ampicillin in *E. coli* chloromycetin in *D. radiodurans*	Lab stock

### RNA Isolation

Cells cultured to OD_600_ = 2 were harvested by centrifugation at 7000 × *g* for 3 min, washed twice in sterile phosphate-buffered saline (PBS; 0.02% KH_2_PO_4_, 0.29% Na_2_HPO_4_ ⋅ 12H_2_O, 0.8% NaCl, 0.02% KCl, pH 7.5), and resuspended in TGY broth to a final OD_600_ = 2 (10^7^ cells/mL). Then, cells were treated at 48°C. These cells were harvested by centrifugation at 12,000 × *g* for 3 min and stored at −80°C. The untreated cells (30°C) were also harvested and stored in the same manner. Total cells from *D. radiodurans* were prepared using TRIzol reagent (Invitrogen, Thermo Fisher, Waltham, MA, United States) with Lysing Matrix Tubes (MP Bio, CA, United States) and total cellular RNA was extracted with the PureLink RNA Mini Kit (Invitrogen, Thermo Fisher, Waltham, MA, United States) following the manufacturer’s instructions. RNA purity was assessed using absorbance readings (260 nm/280 nm) with a NanoDrop^®^ spectrophotometer (Thermo Fisher, Waltham, MA, United States).

### 5′RACE

The transcriptional start site of *DnrH* was determined using the 5′ RACE kit (Roche, Mannheim, Germany) following the manufacturer’s instructions. Briefly, an initial strand of cDNA was generated using a sequence-specific primer (i.e., GSP1) to the *DnrH* gene. The first strand cDNA was purified, and the 3′ end of the cDNA was tailed with dATP by the recombinant terminal transferase. The amplification of dA-tailed cDNA was performed using the sequence-specific primer GSP2 and the anchor primer provided by the 5′ RACE system. The sequence-specific primer GSP3 was using for the second amplification round. Primers GSP1, GSP2, and GSP3, specific for the *DnrH* gene and tested here are listed in Supplementary Table S1. The 5′ RACE products were cloned into the pJET1.2/blunt vector (Thermo Scientific, Waltham, MA, United States) and sequenced to map the 5′ end of the transcript.

### Construction of the Mutant and Complementary Strains

The *DnrH*-mutant strain was generated by replacing the target gene with a kanamycin resistance cassette via fusion PCR recombination, as previously described (Sheng et al., 2005). Briefly, fusion PCR products for the *DnrH* deletions were constructed in two steps. In the first step, PCR was used to generate fragments complementary to the kanamycin-resistance gene from the plasmid pKatAPH3 (920 bp) and the upstream (515 bp) and downstream (529 bp) regions of the *DnrH* sequence using the appropriate primer pairs (Supplementary Table S1). In the second step, the upstream, kanamycin-resistance gene, and downstream fragments were annealed at their overlapping regions and PCR amplified the product as a single fragment using the outer primers (1964 bp). The resulting PCR fragment was directly transformed into *D. radiodurans* and colonies resistant to kanamycin (20 μg/mL) were selected. The mutant was subsequently verified by PCR and DNA sequencing. The successfully constructed mutant was named Δ*DnrH*.

The pRADZ3 vector is generally used for complementation experiments with *D. radiodurans*. This plasmid was digested with *Hin*dIII/*Bam*HI, and the *DnrH* gene was ligated into linear pRADZ3 to generate the complementation plasmid Z3-DnrH. The complementary strain was constructed by transforming Z3-DnrH into Δ*DnrH* and was selected with 20 μg/mL kanamycin and 3.4 μg/mL chloramphenicol. This strain was confirmed by PCR and Sanger sequencing. This successfully constructed DnrH-complemented strain was named *cDnrH*. Similarly, the Hsp20 mutant, complementary strain, and specific point mutants (introduced by site-directed mutagenesis) were constructed using the same method with specific primers (Supplementary Table S1).

### Quantitative Real-Time PCR (qRT-PCR)

Total RNA was extracted using the PureLink RNA Mini Kit (Invitrogen, Thermo Fisher, Waltham, MA, United States) following the manufacturer’s instructions. Then, cDNA synthesis was performed using the PrimeScript^TM^ RT reagent kit with gDNA Eraser (TaKaRa) as described in the manufacturer’s protocol. Subsequently, qRT-PCR was performed using ChamQ SYBR qPCR Master Mix (Vazyme Biotech Co., Ltd., China) with an AB7500 Fast Real-time PCR System (Applied Biosystems, Foster City, CA, United States). The primers are listed in Supplementary Table S1. The 16S (DR_r06) rRNA gene was used as the endogenous reference control to normalize for differences in total RNA quantity, and the relative gene expression was quantified by the 2^–ΔΔCT^ method. Three biological replicates for each condition were conducted.

### Bacterial Growth Curve and Heat Stress Survival Assays

*D. radiodurans* wild-type (WT), mutant (Δ*DnrH*), and complementary (*cDnrH*) strains were grown in shacked (220 rpm) TGY cultures in triplicate at 30°C. The OD_600_ of each sample was measured every 4 h.

WT, Δ*DnrH*, *cDnrH*, and all the other mutant derivatives were cultured in TGY broth with appropriate antibiotics to OD_600_ = 2 at 30°C and were then shifting to 48°C for 4 h. Subsequently, 100 μL of the cell suspension was aliquoted into 900 μL of PBS, after which 10-fold serial dilutions were made for all strains, and 8 μL of each dilution was spotted onto TGY agar plates. These plates were incubated at 30°C for 3 days before colony growth was observed and calculated. The survival rate was expressed as the percentage of the number of colonies in the treated samples compared to those in untreated controls. All assays were performed in triplicate.

### Northern Blot

The northern blot method was used as previously described (Zhan et al., 2016). Briefly, total RNA was isolated from WT and derivatives grown under heat stress and normal conditions. Next, 30-nt single-strand DNA probes were synthesized (Supplementary Table S1), and the 5′ end of the synthesized product was labeled with digoxigenin (Sangon Biotech, Shanghai, China). RNA samples (10 μg) were separated on 8% denaturing gels using 3.2 mL UreaGel 29:1 Concentrate (National Diagnostics, Atlanta, United States), 5.8 mL UreaGel Diluent (National Diagnostics, Atlanta, United States), 1 mL UreaGel Buffer (National Diagnostics, Atlanta, United States), 4 μL TEMED (Thermo Fisher, Waltham, MA, United States), and 40 μL 10% ammonium persulfate (Sigma-Aldrich, MO, United States). The gel was transferred to a nylon membrane using a semi-dry transfer cell and then incubated at 60°C for 1–2 h with freshly prepared cross-linking EDC reagent. The membrane was then hybridized with the suitable digoxigenin labeled probe overnight at 37°C and washed with a low stringent buffer and high stringent buffer. Next, the membrane was incubated in blocking buffer for 3 h at room temperature. Then, the membrane was placed in the blocking buffer with a DIG antibody solution prepared by mixing a DIG antibody solution with blocking buffer at a ratio of 1:15,000 for 30 min. The membrane was washed in DIG washing buffer four times for 15 min each. The membrane was incubated in detection buffer for 5 min and incubated in CSPD solution in the dark for 15 min. Band intensity was analyzed using an Amersham imager 600 RGB (GE, Healthcare, United States).

### Microscale Thermophoresis (MST) Measurements

MST experiments were performed according to a previous report (Jerabek-Willemsen et al., 2011). A set of 30-nt ncRNA oligonucleotides, containing wild-type (wt) or mutated (mut) base-pairing regions of *DnrH* or complementary regions of *Hsp20* mRNA were synthesized by GenePharma (GenePharma, Shanghai, China), as listed in Supplementary Table S2. The wt and mut *DnrH* probe molecules were labeled with 6-carboxyfluorescein. 4 μL of sample containing 200 nM labeled probe and increasing concentrations of a non-labeled competitor (from 18.3 nM to 600 μM) were loaded on standard treated glass capillaries (Monolith NT.115 Series Capillaries, Cat#MO-K002) and measurements were carried out using a Monolith NT.115 instrument (NanoTemper Technologies, Germany) at room temperature in diethylpyrocarbonate water with 40% excitation power and medium MST-Power. The dissociation constants (K_d_) were calculated as previously described (Lippok et al., 2012). Data analyses were performed using Nanotemper Analysis software (NanoTemper Technologies, Germany).

## Results

### Experimental Identification and Transcriptional Start Sites of DnrH

**FIGURE 1:**
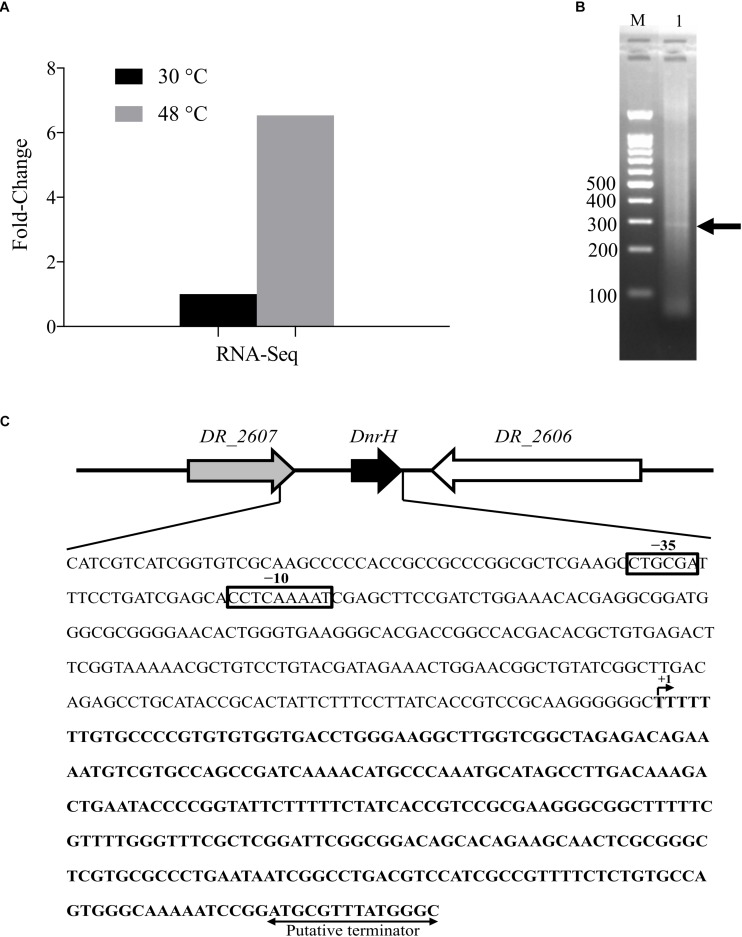
Locus features and the expression of *DnrH*. **(A)** The expression of *DnrH* at 30 and 48°C based on RNA-Seq. **(B)** PCR results of 5′ RACE. The bands are indicated by arrows. Lane M: 100-bp DNA Ladder (Transgen Biotech), cropped from Supplementary Figure S1 lane M; Lane 1: 5′ RACE of *DnrH*, cropped from Supplementary Figure S1 lane 3. **(C)** Physical map and nucleotide sequence of the *DnrH* region of *Deinococcus radiodurans*. Promoter elements (−35 and −10 box) are depicted in solid boxes. ^+1^, transcription start site mapped by 5′ RACE; arrowheads, putative transcriptional terminator.

*DnrH* was identified based on our Illumina RNA sequencing (NCBI database Sequence Read Archive, Accession number: SUB5875813) and a previous report (Tsai et al., 2015). *DnrH* was significantly upregulated (6.54-fold) under heat stress and was selected to elucidate its physiological role in response to this pressure (Figure 1A). We therefore speculated that *DnrH* may positively regulated the tolerance of *D. radiodurans*.

**FIGURE 2:**
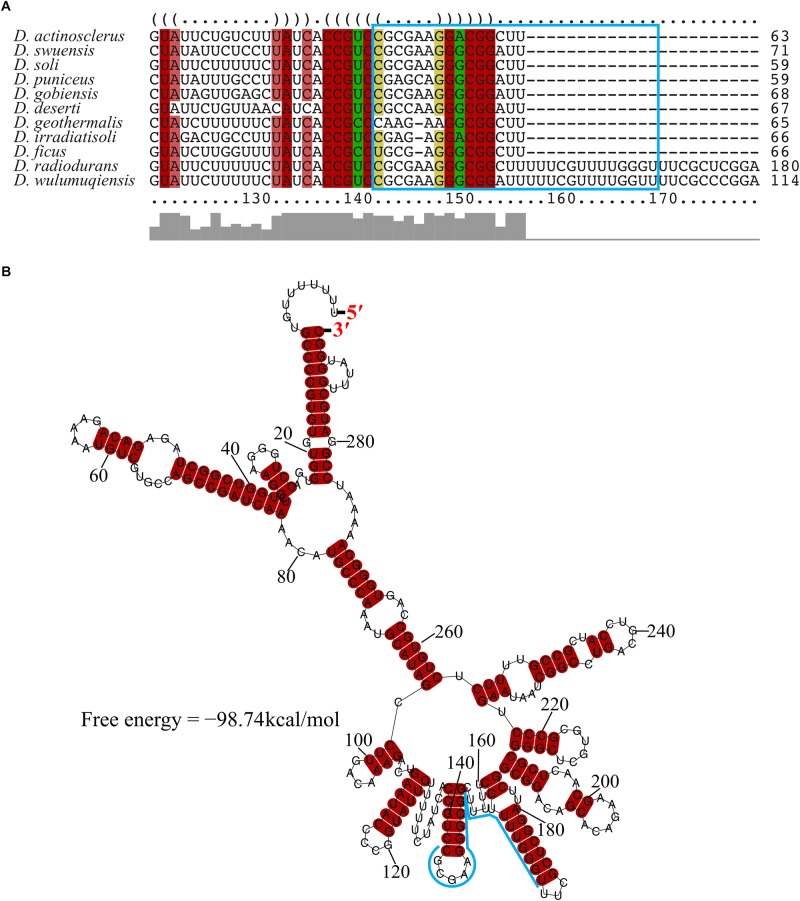
Conservation of *DnrH* sequence and structure. **(A)** Multi-sequence alignment of *DnrH* homolog performed with LocARNA (Will et al., 2012). The colors indicate the structural conservation, for which red indicates 100% of the structural sequence is identical, brown indicates that one nucleotide is changed, and green indicates that two nucleotides are changed. **(B)** Secondary structure prediction performed with RNAalifold (Bernhart et al., 2008). The potential interaction region of *DnrH* is indicated in blue.

The transcriptional start site of *DnrH* was then determined by 5′RACE analysis. The results showed that *DnrH* starts at position 2615872 in chromosome 1, extending 294 nt (Figures 1B,C). It was located in a 490 bp intergenic region between *DR_2606* (encoding a putative primosomal protein N’) and *DR_2607* (encoding a MoaE–MoaD fusion protein) (Figure 1C). The promoter regions (–35 and –10 boxes) were predicted by BPROM (Solovyev and Salamov, 2011) 222 and 191 nt upstream of the transcription start site. Since it is conserved in other Deinococci (Figure 2A), it is speculated that the region must serve the same purpose throughout the genus. Secondary structure alignment revealed that DnrH contains multiple stem loop structures (Figure 2B).

### DnrH Is a Novel Factor Involved in the Heat Stress Response

**FIGURE 3:**
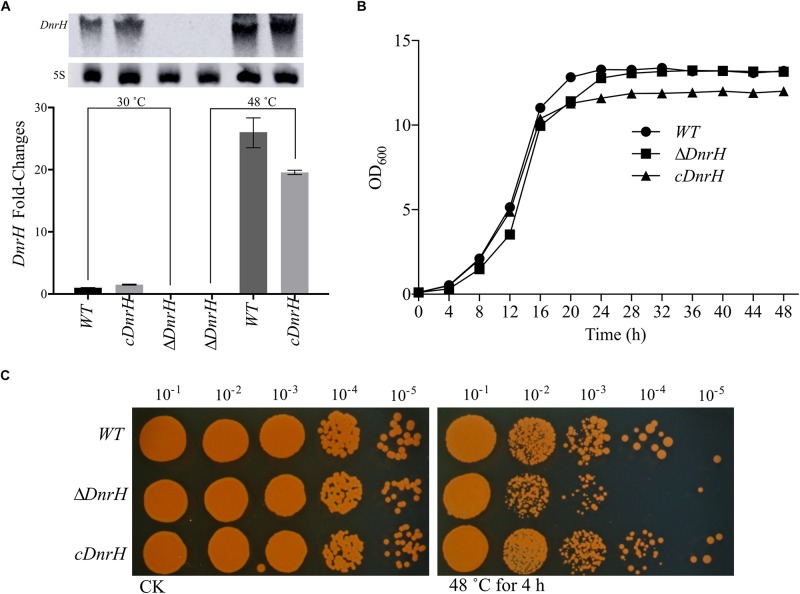
Transcriptional and functional analysis of *DnrH* ncRNA in *Deinococcus radiodurans*. **(A)** *DnrH* transcription under heat stress conditions in the WT, Δ*DnrH* (mutant), and *cDnrH* (complemented) strains. Total RNA was extracted, and the expression of *DnrH* was measured by qRT-PCR. (Inset) RNA northern blot assay using RNA extracted from the same strains under the same conditions and hybridized with the *DnrH*-specific probe. Measurements were normalized to the WT values, and fold differences are plotted. **(B)** Growth curves of WT, Δ*DnrH*, and *cDnrH* in TGY broth. The error bars represent the calculated standard deviation of the measurements of three biological replicates. **(C)** Serial 10-fold dilutions of OD-standardized cultures were spotted on TGY plates after exposure to 48°C.

To further investigate the effect of *DnrH* on heat tolerance in *D. radiodurans*, the *DnrH*-knockout strain Δ*DnrH* and a plasmid-based complementation strain *cDnrH* were constructed. The results of northern blot and qRT-PCR assay showed the significant upregulation of *DnrH* in heat stress response (Figure 3A). The effect of this mutation on the growth potential of the bacterium was examined (Figure 3B). The growth of the mutant did not show any significant difference compared to that of the WT, and the complementation strain showed a slight decrease in growth during the stationary period compared to that in Δ*DnrH* and the WT. This decrease maybe caused by the insert plasmid (Figure 3B). In order to further determine the role of *DnrH* in *D. radiodurans* under heat stress, the Δ*DnrH*, *cDnrH* and WT strains were exposed to high temperature 48°C for 4 h. The relative survival ratios of different strains under heat stress were determined by the recovered colony forming units on TGY plates and showed a higher sensitivity of Δ*DnrH* than the WT under heat stress while *cDnrH* exhibited no significant difference (Figure 3C). These results strongly suggest that *DnrH* act as an important positive regulator in the response to heat stress.

### Hsp20 as Target of DnrH in Response to Heat Stress

**FIGURE 4:**
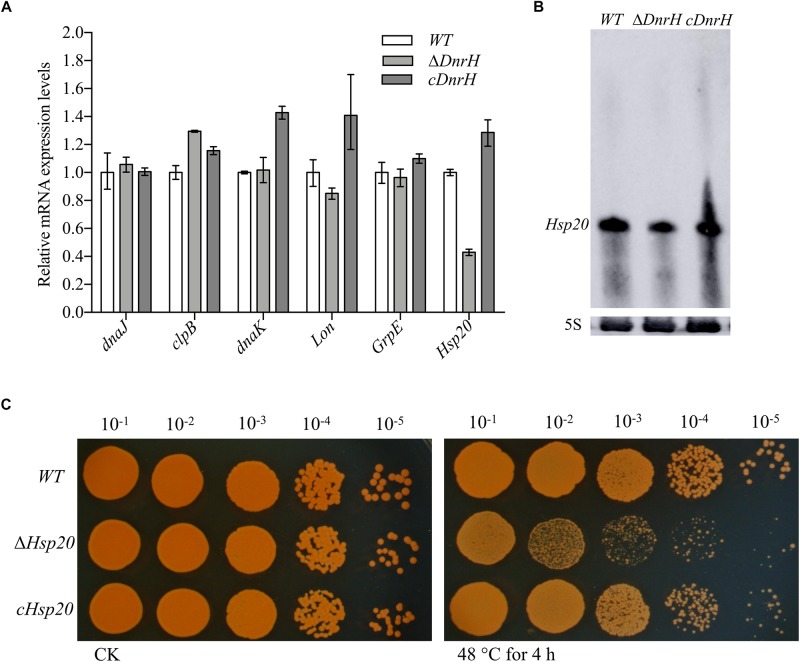
Effect of the presence or absence of *DnrH* on heat-related genes expression and phenotypic analysis of *Hsp20* during heat stress. **(A)** Expression of the most upregulated heat-related genes comparing Δ*DnrH* (mutant) and WT strains. Relative levels of transcripts are presented as the mean values ± SD, calculated from three sets of independent experiments, and normalized to levels in the WT strain. **(B)** RNA northern blot assay using RNA extracted from the same strains under the same conditions and hybridized with the *Hsp20*-specific probe. **(C)** Serial 10-fold dilutions of OD-standardized cultures were spotted on TGY plates after exposure to 48°C.

To identify the potential targets of *DnrH*, we used qRT-PCR analyses to evaluate the transcriptional levels of heat-related genes in *D. radiodurans* (see Supplementary Figure S2). The highly expressed heat-induced genes were selected for further measurements of their relative expression levels in the presence and absence of *DnrH* under heat stress. As shown in Figure 4A, the transcriptional level of *Hsp20* in Δ*DnrH* was significantly decreased compared to that in the WT, whereas its expression in *cDnrH* was consistent with that in the WT strain. This phenomenon was also observed by northern blotting (Figure 4B).

*Hsp20* is described as a molecular chaperone that can prevent the aggregation of denatured proteins during abiotic stresses (Muthusamy et al., 2017). We constructed and tested the *Hsp20*-knockout mutant (Δ*hsp20*) and complementary strain (*chsp20*) and data showed that Δ*hsp20* was more sensitive to heat stress at 48°C than the WT strain, whereas *chsp20* was essentially identical to the WT with respect to this property (Figure 4C). These results indicate that the loss of *hsp20* gene markedly affects heat stress tolerance in *D. radiodurans*.

### DnrH Increases Heat Tolerance in *D. radiodurans* Through Regulation of Hsp20 mRNA

**FIGURE 5:**
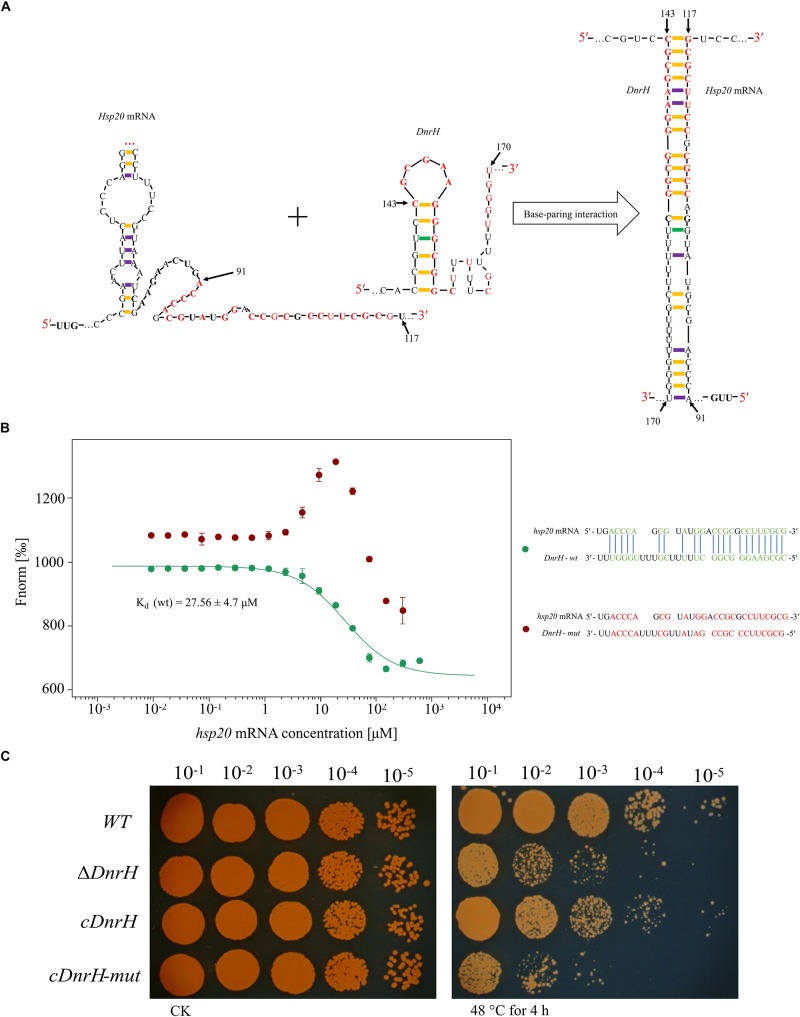
Interaction between *DnrH* and *Hsp20* mRNA *in vitro* and *in vivo*. **(A)** Schematic of the interaction between *DnrH* and *Hsp20* mRNA based on IntaRNA. Exemplary interaction between *Hsp20* and *DnrH*. The region of the interaction is depicted in red bold letters. **(B)** Interactions between FAM-labeled 5′ upstream region of *DnrH-wt* [green, Kd (wt)], *DnrH-mut* (red) and *Hsp20* mRNA. All interactions were measured using Monolith NT.115 and K_d_-values were calculated based on at least three independent replicates using the Nanotemper MO. Affinity Analysis Software. **(C)** Serial 10-fold dilutions of OD-standardized cultures were spotted on TGY plates after exposure to 48°C.

Combining qRT-PCR, bioinformatics analysis, and heat phenotype assay experiments, we preliminarily concluded that *Hsp20* is the target of *DnrH* during the regulation of heat stress tolerance. According to the structure predicted by RNAalifold (see Supplementary Figure S3), *Hsp20* can form a stable secondary structure with -202.77 kcal/mol free energy. In addition, the computational RNA predictive interaction online tool IntaRNA (Mann et al., 2017) revealed that *DnrH* (91–117 bp) can bind *Hsp20* (143–170 bp) with an interaction energy of -14.2573 kcal/mol. The binding sites of the *Hsp20* mRNA are located on a typical stem-loop structure in *DnrH* (Figure 5A). To further validate this, a set of 30-nt ncRNA oligonucleotides containing the wt or mut sequences were synthesized and the interaction between fluorescently labeled *DnrH-wt* or *DnrH-mut* probes and the non-labeled competitor molecule on *Hsp20* mRNA was assayed using MST, which allows for the sensitive measurement of molecular interactions in solution. Results indicated that *DnrH-wt* binds *Hsp20* mRNA at low micromolar concentrations in the titrant, exhibiting a dissociation constant (K_d_) of 28.34 ± 4.7 μM, which suggests a relatively strong interaction. In contrast, the mutant derivative (*DnrH-mut*) harboring substitutions in all complementary bases displayed a complete defect in binding to *Hsp20* mRNA (Figure 5B).

In order to validate this putative *Hsp20* binding sites onto *DnrH*, mutated *DnrH* was cloned into the pRAZ3 plasmid to obtain a recombinant vector (pRAZ3-DnrH-mut). pRAZ3-DnrH-mut was transformed into the DnrH-mutant Δ*DnrH* to construct a complementary strain (*cDnrH-mut*). Heat stress experiments showed that the complementary strain *cDnrH-mut* could not rescue the heat-resistance phenotype (Figure 5C). This result was in agreement with the fact that *DnrH-mut* lost its ability to bind *Hsp20* mRNA. Therefore, we concluded that it is highly likely that *DnrH* enhances heat tolerance in *D. radiodurans* based on a regulatory affect mediated by its 22-nt base-pair complementarity with *Hsp20* mRNA molecules.

## Discussion

The heat response of *D. radiodurans* is considered a classical stress-induced regulatory system that is characterized by extensive transcriptional reprograming. Most current research has led to the discovery and characterization of new heat-related proteins, such as sigma factor, groESL, HspR and DdrI (Airo et al., 2004; Schmid et al., 2005a, b; Meyer et al., 2018). Although much effort has been made to elucidate the molecular mechanisms underlying the tolerance of *D. radiodurans* to heat stress, gene expression reprograming is complex and not yet fully understood. Many ncRNAs have been reported to be associated with bacterial responses to various enviromental stresses (Delihas and Forst, 2001; Majdalani et al., 2001; Masse and Gottesman, 2002; Citartan et al., 2016). While there has been a rapid increase in the identification of bacterial ncRNAs over the last few years, the identification of mRNA targets and the study of ncRNAs’ functions have progressed more slowly. So far, to our knowledge, no ncRNAs with a regulatory role in heat tolerance in extreme bacteria have been reported. Our work shows that *DnrH* could be the first ncRNA with direct roles in regulating heat tolerance in the model extremophilic bacterium *D. radiodurans*. As revealed by qRT-PCR and northern blot analysis, *DnrH* is expressed at a higher level under heat stress, and accordingly, the survival of Δ*DnrH* was reduced in response to heat stress conditions as compared to that with the WT strain.

To fully understand the function of *DnrH* in response to heat stress, the regulatory mechanisms of such factors must be identified. ncRNAs usually regulate other genes at the post-transcriptional level by directly or indirectly base-pairing with the target gene mRNA (Storz et al., 2011; Wagner and Romby, 2015). We identified as target the heat shock protein Hsp20 reported to assist in the refolding and hydrolysis of abnormal proteins (Bepperling et al., 2012). Moreover, previous studies have reported that the expression of *Hsp20* is increased following exposure to various stresses, including temperature changes (Schmid et al., 2005b; Singh et al., 2014). Here we show that the expression of *Hsp20* is significantly down-regulated in the absence of *DnrH* and provide strong indications for *Hsp20* mRNA being a direct target of *DnrH*. Further, results of MST and genetic complementation suggested that *Hsp20* mRNA is a direct target of *DnrH*. The location of this interaction indicated that *DnrH* asserts its positive regulatory effect on *Hsp20* expression by assisting in ribosome binding.

**FIGURE 6:**
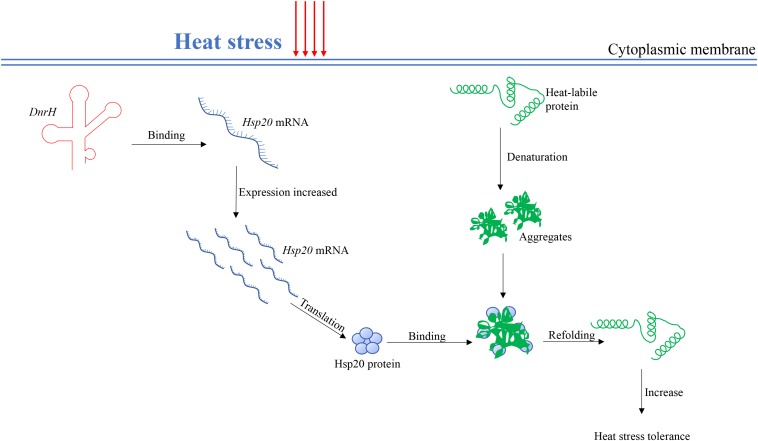
A proposed working model for the *DnrH*-mediated regulatory network in heat stress response in *Deinococcus radiodurans*. *DnrH* act as a riboregulator efficiently binds *Hsp20* mRNA to increase heat stress tolerance. This regulation integrates adaptation to heat stress with other cellular metabolic processes helps to protect cells against heat stress damage. For more details, see the results or discussion.

sHsps can be considered guardians of proteins, especially upon exposure to sudden protein toxic stresses that lead to the accumulation and aggregation of denatured forms. *Hsp20* belongs to the large spherical homo-oligomeric sHsps, which are constitutively active and form stable substrate complexes (Haslbeck et al., 2005). Our data confirmed that *Hsp20* contribute to heat tolerance (Figure 4C), which is consistent with previously reported results (Airo et al., 2004). Further, *in vivo* transcriptional analysis indicated that *Hsp20* transcripts accumulate to high levels during heat stress, again confirming the role of *Hsp20* in high temperature adaptation. A working model, which integrates *DnrH* and *Hsp20* mRNA in response to heat stress is presented in Figure 6. The precise molecular mechanisms underlying the modulation of *DnrH* activities are not yet known and require further investigation. Considering that *Hsp20* functions as a chaperon protein, regulation by *DnrH* might be a two-step process in which *DnrH* regulates *Hsp20* and then affects the transcription of downstream genes. This hypothesis also requires further research.

## Data Availability Statement

All datasets generated for this study are included in the manuscript/Supplementary Files.

## Author Contributions

DX, JW, and ML conceived and designed the study. DX performed the experiment, analyzed the data, and wrote the manuscript. MO and JW analyzed the data and critically revised the manuscript. All authors discussed the results, participated in revising the manuscript, and commented on and approved the manuscript for publication.

## Conflict of Interest Statement

The authors declare that the research was conducted in the absence of any commercial or financial relationships that could be construed as a potential conflict of interest.
