# Supplementary material for: Targeting Hsp20 Using the Novel Small Non-coding RNA DnrH Regulates Heat Tolerance in Deinococcus radiodurans
Source: Front Microbiol. 2019 Oct 11;10:2354. doi: 10.3389/fmicb.2019.02354 (PMC6798082; doi:10.3389/fmicb.2019.02354)
Supplement: Supplementary file 1 [file Data_Sheet_1.pdf]

Supplementary Material

1 Supplementary Figures and Tables

1.1 Supplementary Figures

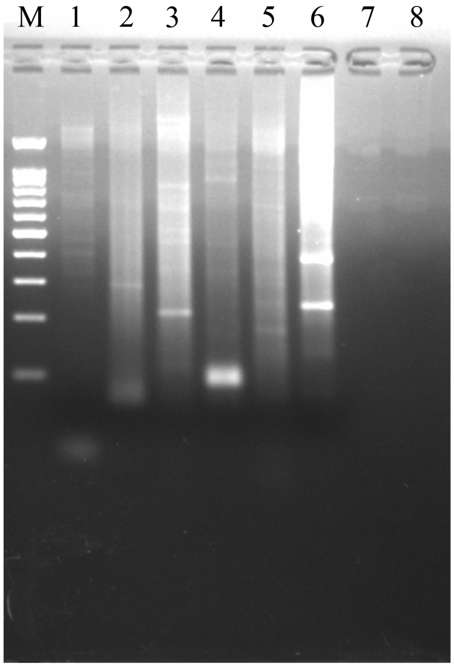

Supplementary Figure S1. The original gels of 5'RACE results. PCR results of 5'-RACE. Lane M: 100bp DNA Ladder (Transgen Biotech); Lane 3: 5'RACE of *DnrH*; Remaining lanes are 5'-RACE results of other ncRNAs those have been done at the same time.

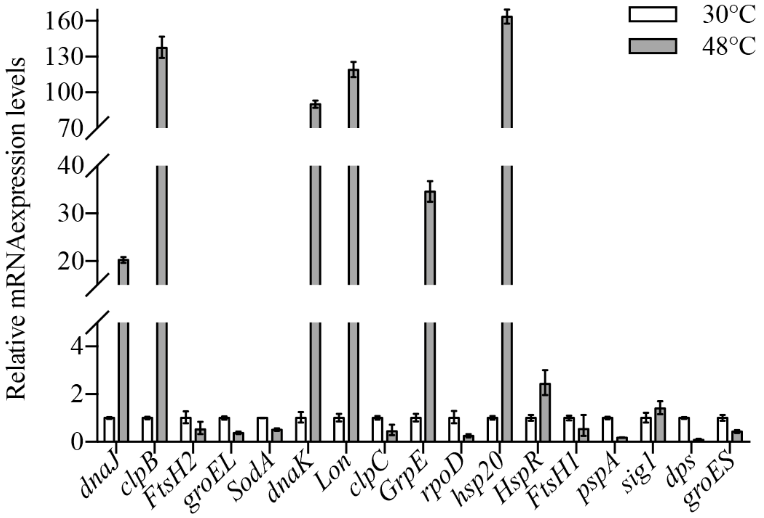

Supplementary Figure S2. Expression of heat regulatory genes in wildtype *D. radiodurans* under heat stress.

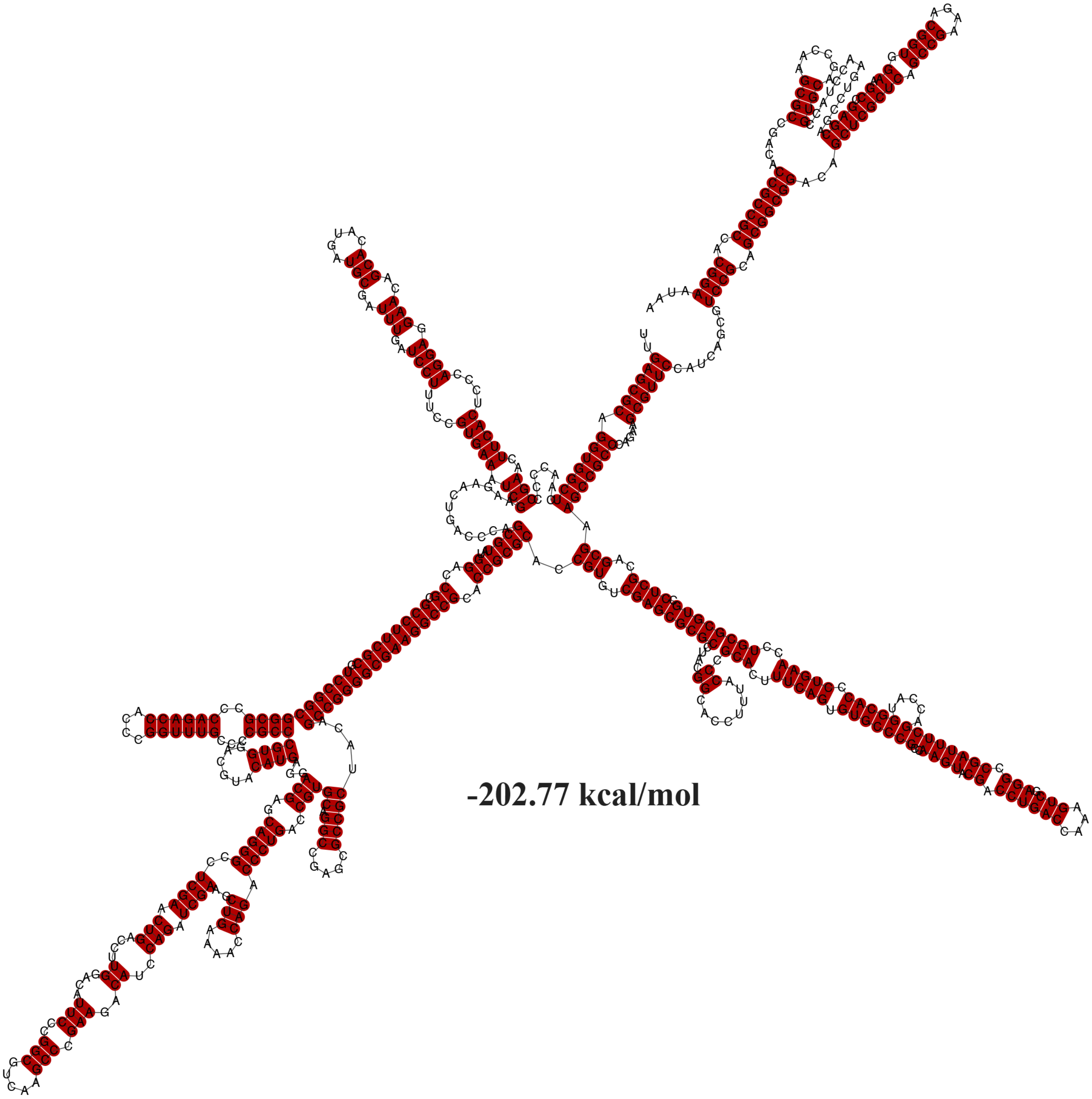

Supplementary Figure S3. Secondary structure of *Hsp20* prediction performed with RNAalifold.

1.2 Supplementary Tables

Supplementary table S1 Premiers used in this study

Primers	Sequence (5' – 3')	Purpose
GSP1	GCCCATAAACGCATCC	5' RACE
GSP2	TCCGGATTTTTGCCCACTGGCACAGA	
GSP3	TTTGCCCACTGGCACAGAGAAAACG	
16S	F: ATTCCTGGTGTAGCGGTG	qRT-PCR
	R: CATCGTTTAGGGTGTGGAC	
DnrH	F: GCGAGTTGCTTCTGTGCTGTC	
	R: GCCTTGACAAAGACTGAATACC	
dnaJ(DR0126)	F: AGGACTACTACGACGTGCTCG	
	R: TGGCCGTACTGGTCGTACAG	
clpB(DR1046)	F: GAACCCTGCTCGACAACGAC	
	R: GCGAGCGTGTCGGCCTTCTG	
FtsH-1(DR0583)	F: AGTTGCTCGTCGAGATGGACG	
	R: AGGTCCACCGACACGTCGAGTG	
groEL(DR0607)	F: GAGCTGGAGGACAAGCTG	
	R: GCCACGGCCTTGTCGATGC	
SodA(DR1279)	F: GCTGGGCGTGGCTGGTCGTC	
	R: GCTTCGAGACTTCGTCCCAGTTC	
dnaK(DR0129)	F: GTCAGGCTGCGCTCAAC	
	R: CGTCGCTCACCAGCTTGCGC	
Lon(DR1974)	F: ACGCACCACGCCACCTGGAC	
	R: TCTGCTCGCGCAGGTAGTACTC	
clpC(DR1117)	F: ACCAAGTACCGCGGTGAATTCG	
	R: TGAGGATGTTGGCTGCGTCG	
ropD(DR0916)	F: TCTCTATCGCCAAGAAGTAC	
	R: TGACGAATCCACCACGTC	
hsp20(DR1114)	F: GGCCTCGAACTGACCTTGGACATTC	
	R: AAAGGTGCCGTAGGCGCGCTCGACA	
HspR(DR0934)	F: TGTCTACGTCATCTCGGTG	
	R: TGCAGGTCGTCGAGCTGGTG	
FtsH-2(DR1020)	F: ATTAAGCTCACCTTCGCGGAC	
	R: ACGAAGTCGGAGCCGGAGATG	
pspA(DR1473)	F: ACCACAAGGACCTCGCCAAG	
	R: TGAAGCCTGACACGCGGTCG	
sig1(DR0180)	F: GTGCAGGCCGCCGAGAGCGAG	
	R: TGCACGTCCGACACGTCGGCCAG	
dps(DR2263)	F: CAAGAGCGAGGCAGCCAGCAAG	
	R: TGATGGTGGTCGCCAGGTTGCGTTG	
groES(DR0606)	F: GCGACCGCGTTCTGGTTG	
	R: GTACTTGGCGAAGTACACG	
5S-dig	F: ACACCCCCGTGCCCATAGCACTGTGGAAC	Northern blot
	R: AAACCCCCGCGCTGACCGACTTTTCCG	
hsp20-dig	F: GGCCTCGAACTGACCTTGGACATTCCCGG	
	R: CACCGTGTCGAGCGCGCCTACGGCACCTTT	
DnrH-dig	F: CGCATCCGGATTTTTGCCCACTGGCACAGA	
	R: CGTTTTGGGTTTCGCTCGGATTCGGCGGAC	

Hsp20-U	F: AGCGTTCAACCTGCGCCTTCTGCGTG	hsp20 mutant construction
	R: CAAGGGCCCTCGGTCTCCATGGTAGTCCTATATTAAAACTTGAGTGC	
Hsp20-M	F: CAAGTTTTAATATAGGACTACCATGGAGACCGAGGGCCCTTGACATTG	
	R: GGTAAAGATGGGTTTACGGGTTATTTGCCGACTACCTTGGTGATCTCG	
Hsp20-D	F: CACCAAGGTAGTCGGCAAATAACCCGTAAACCCATCTTTACCTTTTC	
	R: AGGTCGATGGCGCCGTCGGCGTTCAC	
Hsp20-C	F: ACACTGGCGGCCGTTACTAGTCTGCTCGGGTCGCGCGGCTGG	hsp20 complementary strain construct
	R: TGCCTGCAGGTCGAATCGGATCCTTATTCCGTGGCGGCGGTGTC	
DnrH-mut-com-1	F: CCGAATTCCAGCACACTGGCGGCCGTTACTAGTTACATCGACTACGAAGCC	DnrH site-directed mutagenesis
	R: AATGGGTAAAGCAATATCGGCGGGAAGCGCGACGGTGATAGAAAAAGAATACCG	
DnrH-mut-com-2	F: GCGCTTCCCGCCGATATTGCTTTACCCATTCGCTCGGATTCGGCGGACAGCACAG	
	R: GCTTGCATGCCTGCAGGTCGAATCGGATCCCCCGCGCTCGACCTGAGCGGCAGC	
Z3	F: CTCGCGAGGCCTCGAGATCT	Complementary strain checking
	R: TTGGGTTCTCAGTTTGCTGA	
DnrH-U	F: CCACTGAGCCTCAGCGAGGCCGACGCTTTC	DnrH mutant construction
	R: GCAGTTTCATTTGATGCTCGATGAGTTTTTCTAAGCCCCCCTTGCGGACGGTGAT	
DnrH-M	F: CACTATTCTTTCCTTATCACCGTCCGCAAGGGGGGCTTAGAAAAACTCATCGAGCATC	
	R: GATAAAGAAAGAATAGTGCGGTATACGCGCGTTGTCAACATGGAGACCGAGGGCCCTTG	
DnrH-D	F: GAATCATTCTCAATGTCAAGGGCCCTCGGTCTCCATGTTGACAACGCGCGTATACCGC	
	R: GCTGCTGCGTCAGCTCGCCGAGTGGCAC	

DnrH-C	F: CCGAATTCCAGCACACTGGCGGCCGTTACTAGTTACATCGACTACGAAGCC	DnrH complementary strain construct
	R: GCATGCCTGCAGGTCGAATCGGATCCCCCGCGCTCGACCTGAGCGG	

Supplementary table S2 Synthesized ssRNA oligonucleotide derivatives for MST

Name	Sequence (5' – 3')	Relevant characteristics
DnrH-wt（5'FAM）	CGCGAAGGGCGGCUUUUUCGUUUUGGGUUU	WT, interaction with *Hsp20-wt*
DnrH-mut（5'FAM）	GCGCUUCCCGCCGAUAUUGCUUUACCCAUU	Dismatch mutation, no interaction with *Hsp20-wt*
Hsp20-wt	CUGACCCAGCGUAUGGACCGCGCCUUCGCG	WT, interaction with *DnrH-wt*
